# Art and design workshops at a residential care facility – social care professionals’ experiences of co-creation and participation in designing the physical environment

**DOI:** 10.1186/s12913-024-11851-x

**Published:** 2024-11-07

**Authors:** Ewa Wikström, Synneve Dahlin-Ivanoff, Maja Gunn, Qarin Lood

**Affiliations:** 1 Institute of Management and Organisation, Centre for Health Governance, School of Business, Economics and Law, University of Gothenburg, Gothenburg, Sweden; 2 Institute of Neuroscience and Physiology, Department of Psychiatry and Neurochemistry, Sahlgrenska Academy, University of Gothenburg, Gothenburg, Sweden; 3 Institute of HDK-Valand – Academy of Art and Design, University of Gothenburg, Gothenburg, Sweden; 4 Centre for Ageing and Health, AgeCap, University of Gothenburg, Gothenburg, Sweden

**Keywords:** Art and design workshop, Social care professionals, Nursing home, Co-creation, Participation, Deliberative practice, Developing practice, Sweden

## Abstract

**Background:**

Co-creation and participation among different user groups have been highlighted as pivotal for improving residential care facilities for older persons. However, more knowledge is needed on methods aiming to overcome challenges in constructing inclusive co-creation and participation processes. In particular, there is a need for knowledge concerning how art and design workshops could contribute to co-creation and participation of social care professionals designing the physical environment in residential care facilities. Therefore, this study aimed to examine how art and design workshops can contribute to co-creation and participation in designing the physical environment with residential care facility professionals.

**Methods:**

A qualitative method was used to investigate social care professionals’ experiences. Data were collected through semi-structured group interviews and analysed with inspiration from Corbin and Strauss’s analysis method.

**Results:**

This study concludes that art and design workshops could serve as deliberating and developing practices. First, the art and design workshop as a deliberating practice involves conditions that create a communication arena and space supporting professionals in sharing experiences and voicing different perspectives. Second, the art and design workshop, as a developing practice, supports shared agency through dialogue focusing on designing the physical environment in residential care facilities, using photographs, materials, and fabrics.

**Conclusions:**

This research contributes to the understanding of the relevance of art and design workshops and co-creation between artists and social care professionals in designing the physical environment in residential care facilities. The study can be valuable in identifying important mechanisms that facilitate co-creation and participation among social care professionals, as well as the development of art and design as a tool for improving environments in residential care facilities. The research focuses on how art and design workshops could influence co-creation and participation through art and design from the perspective of social care professionals.

**Supplementary Information:**

The online version contains supplementary material available at 10.1186/s12913-024-11851-x.

## Background

Co-creation, participation and inclusive research methods among different user groups have been highlighted as pivotal for improving residential care facilities for older persons (see, for example [1]) and for involving older persons in developing service practices (see, for example, [2]). However, the complexity and challenges of designing supportive models and methods for such participation are significant [3–6]. This study is part of a larger research project with the overarching aim to artistically design a residential care facility by collaborating in the planning, construction, and furnishing process, and to evaluate the importance of art and design for older persons in terms of social engagement and sustainable living environments. With focus on the experiences of social care professionals within the studied setting, the aim with the study was to examine how art and design workshops could contribute to co-creation and participation in designing the physical environment with residential care facility professionals (the experiences of older persons are explored in a separate study). Through the aim, we will shed light on the research question: If and how did art and design workshops contribute to the co-creation and participation in designing the physical environment of social care professionals working in residential care facilities?

The United Nations’ strategic policy and action plan, Agenda 2030, highlights inclusion and participation as essential for developing sustainable societies and communities. However, there is a lack of knowledge on how to build physically and psychosocially sustainable, adaptable, and inclusive housing for older persons, incorporating sustainable practices that promote social values. Specifically, very few scientific studies have evaluated the process of using art and design to co-create living environments, originating from the perspective of residential care facility professionals [7–10]. Drawing on the core principles outlined by Sanders and Stappers [11, 12], a goal with this study was therefore to acknowledge the role of art researchers as facilitators for co-creative processes, supporting participants to tap into their own knowledge and creativity. As described by Sanders and Stappers [11], co-creation could unlock tacit knowledge and involve participants in ways they find meaningful and engageing [11]. With focus on the role of generative tools and creative pratices, co-creation could thus enable participants to access different forms of knowledge and collectively visualise and design physical environments [12].

Residential care facilities for older people should not only be safe places to live but also places where people can thrive and feel good at the end of life. A problem is that residential care facilities for older persons serve as both a home and a care environment. Health and the ability to make meaningful choices are crucial resources for dealing with changes in everyday life. For older persons, this entails being able to manage daily activities at home and participate in society [13, 14]. For staff, it is about meaningful work and fostering a sustainable work environment [4]. For organisations, it is about creating sustainable processes where people have a say under sustainable conditions [14]. Being able to participate in activities on their own terms, either alone or together with other people, is expected to enhance the health and well-being of older persons in the residential care setting, fostering a so-called person-centred climate [15]. The concept of a person-centred climate has been developed to describe people’s overall perception of environments where care takes place. It is defined as a place, space or context perceived as person-centred, based on the dimensions of safety, familiarity, and hospitality. Thus, it involves both safe care and an everyday environment where people feel welcomed [15, 16].

In a study on health professionals’ involvement in research on ageing and health, Laustsen et al. [17] highlight positive results related to knowledge integration and learning that might be beneficial in developing an improved environment in the care of older persons. However, they also highlight some challenges related to both practical and methodological issues. The practical issues concerned a lack of resources, such as time and funding for professionals to participate in the knowledge integration and learning process. The methodological issues were related to the design of the method for participation, for example, the language embedded in the method, and difficulties in translating scientific concepts and language to facilitate participation of professionals [17]. Practical and methodological challenges in user and professional participation have also been highlighted in other earlier studies on ageing and health (see, for example, [18–21]). Thus, earlier studies on co-creation and participation of professionals call for further research within the field of ageing and health.

The physical environment of a residential care facility holds both existential and social meanings, influencing both how people feel and what they do [15, 16], while art can aim to have an aesthetic function and stimulate a meaningful context [21–24]. Based on mutual respect and the sharing of ideas, we have therefore applied an art and design method for co-creation and participation to give voice to all participants, with the goal of increasing the relevance of research findings to the target groups. The decision to apply these methods stems from their inherent emphasis on creativity, inclusivity, and iterative exploration, which we believe are crucial for engaging participants from diverse backgrounds. These methods are especially effective in fostering an environment where all participants—regardless of expertise or experience—can contribute equally. By using visual and creative tools, art and design methods allow for more nuanced forms of expression, particularly for participants who might find traditional methods (such as interviews or surveys) restrictive. Moreover, these approaches encourage the co-creation of knowledge, enabling participants to actively shape the research process. This participatory nature ensures that the findings are more directly informed by the lived experiences and perspectives of the target groups, which increases the relevance and practical applicability of the research outcomes. This approach not only enhances the authenticity of the findings but also facilitates the integration of research into practice, as it resonates more deeply with the end users’ needs and realities. We refer to art and design methods as creative and reflective practices that allow participants to express and communicate their personal experiences; being exploratory; facilitating open-ended dialogue; generating solutions; visualizing practical changes; and more. By employing art and design methods, the study enables participants to engage in co-creation at both a reflective and practical level, ensuring that their experiences are fully integrated into the research process and that the results are actionable in practice. These are exploratory and often subjective, aimed at facilitating open-ended dialogue. In contrast, design methods refer to the structured processes of generating solutions and visualising practical changes, often focusing on specific outcomes like the improvement of care environments. By employing both art and design methods, the study enables participants to engage in co-creation at both a reflective and practical level, ensuring that their experiences are fully integrated into the research process and that the results are actionable in practice. Such co-creation is also suggested as a way to improve the possibilities for knowledge integration and learning, or to translate evidence into practice and implement research-based knowledge in day-to-day practice [18–21]. Therefore, representatives from a residential care facility, i.e. older persons, and social care professionals, will be involved in testing the art and design method during workshops in a residential care facility setting. This will enable the participating organisation to meet the needs of older persons and professionals, in line with the environment and resources available. This study focuses on the professionals, and the potential of using art and design workshops for the co-creation and participation of professionals in designing the physical environment.

## Co-creation and participation of professionals

The development of knowledge through co-creation and participation expands the space for diverse perspectives and judgments by actively shifting decision-making power and influence to all participants, ensuring that their input shapes the outcomes of the process [11]. Earlier research shows that a development process involving distributed forms of co-creation of social values through partnerships or collective forms of leadership can take place through collective and broad forms of responsibility, control, and influence [25–27]. The development of knowledge through co-creation and participation expands the space for diverse perspectives and judgments by actively shifting decision-making power and influence to all participants, ensuring that their input shapes the outcomes of the process [11].

The development of a model for art and co-creation can increase the democratic basis for a broader influence for all people involved in the co-creation of a physical and social living environment [28], especially for professionals closest to the older persons [29]. While care services increasingly embrace co-creation to accommodate diverse needs and improve outcomes [30, 31], this approach is still developing and holds great potential for enhancing the democratic influence of all stakeholders, particularly those closest to the older persons. Distributed forms of co-creation focus on increasing and broadening influence and agency through interpersonal interactions, rather than formal roles and responsibilities [32]. When co-creative practices emerge in a constructive and trusting manner, they can develop into a shared agency [33] for sustainable residential care facilities.

In Sweden, there are local initiatives to work and organise residential care facilities in ways that increase influence among health and social care professionals, which indirectly also enhance the influence of older persons and relatives [34]. When such a process takes place with trust, commitment and joint responsibility, the development work can also be strengthened among managers and professionals, which, in turn, provides older persons greater scope for influence over care, service, and the design of the environment [29]. However, Sweden currently lacks structured models for co-creative processes that foster trust among participants. It is our intention to contribute to the development of such a model, utilising art and design methods along with continuous dialogue in art and design workshops.

Previous literature [34, 35] suggests that, when planning construction and launching activities characterised by co-creation, arenas for communication, interaction and meaningful participation should be central to both the physical construction and the activity and organising practices [34, 35]. Previous research also indicates that a home-like environment in residential care facilities is not only about the physical design of the home but also about social values and psychosocial aspects of what happens there [15, 16]. The environment of residential care facilities holds both existential and social significance and affects how people feel and what they do. For example, the environment can promote well-being, facilitate community for older persons, and contribute to pride and well-being for the professionals. The thriving theory as described by Bergland and Kirkevold [36] provides a framework for understanding how older persons in residential care facilities experience the best possible life in relation to the physical and psychosocial aspects of the environment in which they live [36]. Thriving is also about accepting the residential care facility as one’s home, feeling positive, supported and cared for while retaining independence and balancing one’s freedom to be alone and to be with others [37].

In addition to the benefits for those participating in forms of social values, such as well-being, satisfaction, engagement, and influence; transferability, methods of co-creation, such as art and design workshops, should be replicable in other contexts. From a workplace perspective, art and design workshops may represent an opportunity to develop innovative forms of participation through distributed forms of co-creation (see, for example, [35]). The aim is to develop residential care facilities that integrate social values, such as well-being, satisfaction, commitment and influence among older persons and social care professionals to meet the older persons’ needs and to create a sustainable working environment. From an organisational and societal perspective, co-creating the care environment by involving older persons and social care professionals aligns with national guidelines on value-based care that emphasise meeting the diverse needs of older persons [38, 39]. It can also be viewed as a way of supporting staff retention by strengthening the attractiveness of working in care.

When it comes to different forms of co-creation, models have been developed in England, Australia, and Denmark to strengthen co-creation in care activities (e.g. Shared Governance Programme and Health LEADS Australia). Follow-up studies of these models have shown effects such as improved outcomes and core activities, more commitment among care professionals, improved development work, and indications of more attractive work environments [40, 41]. In Australia, results from an action research project where co-creation was developed in care for older persons indicated that the most critical aspect of developing forms of co-creation was creating physical arenas and communication processes to enhance understanding, sense-making, and interaction. By continuously articulating and sharing insights and experiences, the conditions for co-creation could be developed and improved. The development of distributed forms of co-creation can and should thus be seen as an ongoing process and method with a focus on influence and participation in the public sector [35].

## Methods

The project forms part of GoCo Health Innovation city, a platform facilitating diverse collaborations for innovative solutions. Specifically, the larger research project involves a multidisciplinary partnership between researchers from the Centre for Aging and Health (AgeCap) at the University of Gothenburg, and “Vectura Fastigheter AB”, a company specialising in community property development, including residential care facilities. The project unfolds in two phases: first, an exploration of the planning construction, and furnishing processes; and second, an evaluation of the artistic interventions employed. This study is part of the first phase.

### Study design

This study uses a qualitative design to emphasise the use of art and design workshops, including both an artist and social care professionals. This design is based on the purpose of the study, which is to examine how art and design workshops can contribute to co-creation and participation in designing the physical environment with residential care facility professionals. The art and design workshops were organised around several activities, and different forms of visual prompts such as photographs, materials, and fabrics.

### Art and design workshops

In this study, co-creation and participation were facilitated through art and design workshops, which involved both the artist and social care professionals. Utilising collage-making, visual mapping, and material prototyping as core methods, art-based methods were employed to foster reflection and open discussion, enabling participants to express their experiences and insights visually. Material prototyping was also used as a design-oriented method to involve participants in creating tangible representations of care environments using fabrics, photographs, and other materials. This method served to translate abstract ideas into concrete proposals for changes in the physical environment of residential care facilities for older people.

The artist (MG) started the workshops with the social care professionals by describing the aim of the workshops at the studied care facility with an emphasis on both the process and the potential outcomes. MG communicated that the primary goal was to engage in a co-creative process where their input would play a crucial role in shaping the design of the residential care facilities. The collaborative nature of the workshops was emphasised, highlighting the importance of the professionals’ expertise and perspectives in achieving meaningful outcomes. Then, the artist presented photographs of different examples of houses in different contexts to the participating professionals. The activities were introduced with questions such as: From your perspective as professionals, what are your experiences of how older persons want to live? When looking at the photographs, topics such as colours, materials, lighting, and furniture were discussed. The workshops also included looking at technical drawings of furniture, where the professionals discussed their preferences for the appearance of chairs or sofas. They were also invited to add their own colours to the drawings. Thereafter, professionals were presented with a variety of fabrics to discuss characteristics such as material, softness, patterns, and colours. Finally, they were asked to draw pictures depicting how they wanted the facility to look.

### Participants and procedure

Participants were strategically selected based on specific criteria to ensure a diverse and representative group of professionals with relevant experience. The parameters included their roles within the social care environment, level of experience, and direct involvement in the daily care of older persons. Our aim was to involve a mix of professionals who could provide a range of insights into the co-creation process, including those with direct caregiving responsibilities and those involved in the broader management of care environments.

The data collection was conducted through group interviews following Corbin and Strauss’s [42] qualitative method approach, which is known for studying processes and actions within particular contexts. The group interview method was used to get a deeper exploration of perspectives on targeted questions across different groups [43, 44]. Two group interviews were conducted with a total of eight assistant nurses, with four participants in each group. In addition, a student studying to become a registered nurse participated in the second group interview. The length of work experience among the assistant nurses ranged from one month to 10 years within the studied residential care facility. For an overview of the work experience of participants, please see Table 1.

**Table 1:** Work experience of participants

**Group 1**	
Nursing assistant (D)	Has worked in residential care facilities for eight years. Works a lot with food, meals, and culture.
Nursing assistant (S)	Has worked in residential care facilities for nine to ten years and three years in the studied residential care facility. Is a cultural and documentation officer.
Nursing assistant (F)	Has worked in residential care facilities for eight years and is an activity coordinator at the studied residential care facility.
Nursing assistant (R)	Has worked in residential care facilities for twelve years. Has worked at the studied residential care facility for ten years.
**Group 2**	
Nursing assistant (B)	Has worked in residential care facilities for two years, and for three months in the studied residential care facility.
Nursing assistant (O)	Has worked for 10 months at the studied residential care facility.
Nursing assistant (G)	Has worked for one month at the studied residential care facility. Is a care supervisor and specialist assistant nurse in dementia and welfare technology.
Nursing assistant (S)	Has worked in residential care facilities for about 15 years, 10 years at the studied residential care facility.
Student (I)	Is a student studying to become a registered nurse.

Participants in this study were not randomly selected but rather strategically chosen because of their relevance to the research question [44]. The group interviews revolved around the concept of housing, i.e. examining what a physical environment means with a homelike, personal, and engaging living environment for older persons, as well as exploring forms of co-creation linked to the physical environment.

The group interviews were conducted one week after the co-creation workshop. The question areas for these interviews were:

1. *Experiences of social care professionals regarding the co-creation between an artist and social care professionals.* Example of question: “Can you describe your experience of working with the artist during the co-creation process?“.
2. *Experiences of social care professionals regarding the results of the co-creation that took place.* Example of question: “What are your thoughts on the outcomes of the co-creation?“.
3. *Reflections and overall comments by social care professionals.* Example of question: “Do you have any reflections or overall comments on the co-creation process?”

The interview used in our study was developed for this study. The interview guide is attached as a supplementary file.

### Data analysis

The interviews were recorded, transcribed, and analysed in parallel with the data collection, inspired by Corbin and Strauss’s [42] analysis method. The aim of the chosen method and study design has been to focus on art and design workshops involving both artists and social care professionals to support co-creation, participation and the sharing of experiences.

Initially, we labelled statements in the interview transcripts and categorised the descriptive content, focusing on patterns of similar statements and their connections. An example of initial codes concerned how the art and design workshop was structured. Certain patterns were interpreted when participants answered similarly or used similar examples, while at other times, they did not. For the first-order coding, the first author (EW) identified and mapped out code words that were used in a chart to gain an overview of each interview and to draw parallels between them. Thereafter, the empirical categories were organised based on the concepts of co-creation and participation. For the second order coding, we looked at statement patterns and the connections between them through the lenses of co-creation and participation, specifically focusing on what characterises:

A) *Arenas for communication* and conditions supporting *increased space for different perspectives and judgements* by releasing and shifting real influence on all involved parties. This involves interaction and meaningful participation to increase and broaden influence and agency through interpersonal interactions, rather than formal roles and responsibilities.
B) *Shared agency* for improving the physical environment at residential care facilities.

## Results

Supporting co-creation and participation in designing the physical environment of residential care facilities, the analysis reveals two important functions of using art and design workshops: a deliberative practice and a development practice. As a deliberative practice, the art and design workshops support social care professionals in sharing experiences and expressing different perspectives. In contrast, as a developing practice, these support shared agency and an inclusive dialogue, focusing on developing the physical environment in residential care facilities using photographs, materials, and fabrics. The next two sub-sections describe and comment on these practices and an overview of the results is presented in Table 2:

**Table 2:** Overview of the results

Arenas for communication and space for different perspectives and judgements: Experiences of social care professionals regarding art and design workshop as a deliberative practice	Shared agency: Experiences of social care professionals regarding art and design workshop as a co-created development practice
a) Creation of space for different perspectives and judgements b) The role of artistic material and activities as deliberative mechanisms for co-creation and participation c) The role of art and design workshop in raising awareness of taken-for granted prioritisations among the professionals d) The role of art and design workshop to focus on listening to each other’s experiences	• Well-organised group arrangements for professionals, including materials and activities that enable those involved to share experiences and listen to each other • Practical solutions and the possibility to use solutions in everyday environments and work settings • Capability of professionals to use older people’s experience to develop the physical environment • The materials and activities within the art and design workshop facilitate shared agency • The uniqueness of the method in creating shared agency and development

### Arenas for communication and space for different perspectives and judgements: art and design workshops as a deliberative practice

The first function of art and design workshops in the co-creation and participation of professionals requires extensive communication between the professionals, focusing on voicing their different experiences: a deliberative practice.

The findings show that arenas for communication and space for different perspectives and judgement are characterised by a deliberative practice involving four intertwined aspects:

- Creation of space for different perspectives and judgements.
- The role of artistic material and activities as deliberative mechanisms for co-creation and participation.
- The role of art and design workshop in becoming aware of taken-for granted prioritisations among the professionals.
- The role of art and design workshop to focus on listening to each other’s experiences.

The next four sub-sections describe and comment on these practices.

#### Creation of space for different perspectives and judgements

When it comes to the professionals’ experiences with co-creation and participation in art and design workshops, they expressed several positive experiences that were partly associated with how the method created space for their own reflection. They also described that the method created space for active participation through input on changes in the physical environment within the studied residential care facility. Co-creation was deemed beneficial, with the professionals’ experiences being utilised to make the environment attuned to the needs of older persons. The workshops also held significance because they directly affected the professionals’ working environment. For instance, the design of the environment, particularly the hard floors, had caused discomfort and leg and knee issues for the professionals. An example of how the professionals described the benefits of the workshops is given in the following quote:

*I thought it was fun with the co-creation because you got to reflect on the actual work environment here. How do we feel about the colours? What about the work environment? I spent a lot of time thinking about whether we have the right colours*,* and if we have the right furniture? Are they adapted to the care recipients? (Nursing assistant*,* S)*

#### The role of artistic material and activities as deliberative mechanisms for co-creation and participation

Artistic material and activities, such as drawing pictures, were described as catalysts for creativity, as deliberative mechanisms for co-creation and participation. The professionals initially focused on their own perspectives, but as the artist and the other professionals engaged in the workshop, they also considered the needs of older persons. For instance, several professionals emphasised the importance of nature for the older people. This concerned opportunities for them to sit indoors and look out at nature, but also about having plants indoors and being able to go out for a walk easily with the older persons. The workshops supported the professionals in incorporating nature into the environment, especially when direct access to outdoor environments was not feasible.

The professionals also described that the workshops centred on discussions surrounding the images of rooms presented by the artist. By engaging in exercises involving visual stimuli, the professionals could examine various images and provide feedback regarding colours, etcetera. This was viewed as an enjoyable way to learn about the thoughts and emotions evoked in others, and it stimulated their creativity. The images provided by the artist depicted potential designs for a residential care facility. They also inspired the professionals with fresh ideas and insights into what a residential care facility could look like to accommodate the needs of the older persons. For example, one professional said:

*It was a good exercise because when you have something to talk about and develop it further and think if you do this*,* for example*,* use a different colour or draw curtains for windows at night*,* then it will be like this. It is easier to think further when you have a picture to start from. All elements of the exercise worked well. (Nursing assistant*,* E)*

#### The role of art and design workshops in raising awareness of taken-for granted prioritisations among the professionals

It was highlighted that the workshops made the professionals realise that they tended to focus on the older persons who need the most help. They described that they found it difficult to attend to various older persons and often chose to prioritise those with the greatest need for care. This was expressed, for example, when the staff examined and discussed the fabrics provided by the artist during the workshop. However, the workshop gave them the opportunity to re-evaluate this approach, and they expressed that they will now try to think more about the needs of slightly more alert older persons who also live there.

The professionals expressed their desire to continue using art and design workshops to promote co-creation and participation among social care professionals in residential care facilities. The role of such workshops in raising awareness of taken-for granted prioritisations among professionals is illustrated by the following quote:

*I only thought about myself in the beginning. But the more my colleagues talked*,* and the artist talked*,* I started to think about the tenants’ needs. (Nursing assistant*,* F)*

#### The role of art and design workshops to focus on listening to each other’s experiences

The professionals realised that they have a lot to learn from each other and acknowledged the importance of listening to each other’s experiences and opinions. Taking the time to sit down and listen to others’ perspectives was described as valuable, prompting them to consider aspects they had not previously contemplated. The workshops functioned as an arena for discussion, shedding light on the rationale behind their actions and providing clarity regarding the purpose behind the practices of different professionals. This is visualised in the following quote:

*I thought it was really fun to sit down and listen to everyone else and what they think and feel. It makes you think about things that I haven’t considered before. For example*,* there are some staff who close curtains in the evening because some older persons are afraid of reflections when it’s dark. I hadn’t thought about that. I can benefit from that because now I understand why they do this*,* and I hadn’t thought about it. (Nursing assistant*,* E)*

The professionals highlighted how the art and design workshops functioned as a platform for communication, providing a space to discuss and listen to each other. By focusing on the different experiences of the professionals within residential care facilities, the use of diverse materials, photographs and artistic techniques was important for voicing their experiences within a context of safe and trust-based dialogue.

### Shared agency: experiences of social care professionals regarding art and design workshops as a co-created development practice

The professionals highlighted that workshops could also serve as a development practice. For example, the materials, photographs, and the dialogue during the workshops were described as facilitating conditions in the effort to achieve shared agency through co-creation and participation among the social care professionals. This required the concretisation of practical examples of developments in the environment. The professionals experienced that the activities during the workshop reminded them of the importance of thinking outside the box when developing the environment at the residential care facility.

The findings demonstrate that shared agency for developing the physical environment at residential care homes, based on inclusive co-creation and participation involving social care professionals, is characterised by:

- Well-organised group arrangements for the professionals, including materials and activities that enable those involved to share experiences and listen to each other.
- Practical solutions and the possibility to use solutions in everyday environments and work settings.
- Capability of professionals to use older people’s experience to develop the physical environment.
- The materials and activities within the art and design workshop facilitate shared agency.
- The uniqueness of the method in creating shared agency and development.

The next five sub-sections describe and comment on these practices.

#### Well-organised group arrangements for professionals, including materials and activities that enable those involved to share experiences and listen to each other

The professionals described a need for group arrangement to create a shared agency, including using materials and activities to share experiences and listen to each other’s experiences with art and design. By facilitating the expression of different experiences and values in the design of residential care facilities, the workshops provided the professionals with opportunities to engage with various materials and gain insights from diverse perspectives. Being part of a cohesive group, with shared objectives, the professionals considered both the needs of the older persons and their own needs as professionals. Engaging in the workshops was viewed as an approach to become aligned with each other’s experiences and to prioritise shared goals. This is described in the following quote:

*It was a good group. We had the same goals. We thought about the needs of the older persons and our needs. That everyone thinks about the needs of the older persons and safety*,* sound*,* lighting*,* and materials. Talking about it - I think that’s important. (Nursing assistant*,* F)*

#### Practical solutions and the possibility to use solutions in everyday environments and work settings

The professionals described how the workshops generated numerous ideas on solutions to everyday issues. They also felt that they could conduct art and design workshops with the older persons to develop the environment. Moreover, they highlighted that these workshops initiated thought processes, leading the staff to think about how they could be more ‘open minded’ concerning how to design the environment. For instance, there was a painting room at the facility that could be used for their continued work with art and design workshops, allowing them to carry out exercises with colours and materials with the older persons. Shared agency, based on practical solutions that could be used in the everyday environment, was described in relation to creating pleasant sounds, avoiding accidents, maintaining hygiene and alleviating neuropsychiatric symptoms, as depicted by the following quote:

*Many things we knew already; we are very advanced here. Wooden blocks can be used to reduce anxiety*,* but they must be easy to clean and not disturb others. There were some materials that we had not seen before. You think it’s good stuff*,* but it must be easy to clean or easy to put on and take off. Hygiene is important. (Nursing assistant*,* F)*

#### Capability of professionals to use older people’s experience to develop the physical environment

The professionals emphasised that the workshops sparked them to think in a different way when developing the environment. For example, they said that they would now think differently when ordering furniture, paying attention to colours and textiles. They expressed that they would place orders together, ensuring that professionals from various departments are involved in the decision-making when ordering. Accordingly, the professionals emphasised that this method could be used not only to share their experiences but also to involve older persons in co-creation. Specifically, they mentioned the importance of using tangible materials in the co-creation and participation process, such as using fabrics or bringing in a picture or a piece of wood to relate to. They concluded that improvements do not have to cost so much and emphasised that older persons are often very wise if you, as a social care professional, take your time to sit down with them and open your ears and eyes, to listen to both what they say and what they show with their behaviour and feelings, as reflected in this quote:

*Now*,* I sit here and smile. I think about us as staff. Are we capable of thinking outside the box. The older persons with dementia are often outside the box all the time and do things that we would never think of them doing. But those of us who work with them and see them*,* are we able to think outside the box? Do we manage to think through what a residential care facility should look like*,* so that both I as a staff member will be happy*,* and the residents will also be happy. (Nursing assistant*,* G)*

#### The materials and activities within the art and design workshops facilitate shared agency

The professionals pointed out that the workshops resulted in them realising that they can use co-creation to stimulate the older persons. They noted that shared agency could be facilitated by using materials (photographs, fabrics, and paintings) and through activities conducted in the art and design workshop. Fabrics were also identified as beneficial for older persons, in terms of them offering a sense of comfort. This could mean fabrics that provide warmth and a sense of security for older persons with cognitive impairments. Inspired by the workshops, the professionals discussed ways to utilise the fabrics available for use, for instance, repurposing old fabrics that might evoke memories for the older persons.

One key lesson highlighted by the professionals was that shared agency could be strengthened by involving older persons who may have a better ability to innovate than the professionals. They described the importance of developing the physical environment in a manner that supports the older persons to connect with their feelings and thoughts. Additionally, they acknowledged that they often think about the environment at the residential care facility from a nursing perspective, rather than considering the perspectives of the older persons themselves. As social care professionals, they recognised that it was easy to forget that it is possible to ask the older person directly. Consequently, they thought it was a good idea to involve the older persons in art and design workshops. For instance, the older persons may like certain materials, such as glittery or patterned fabrics, whereas the professionals may think about the environment from a more practical perspective.

The artist’s way of working with various materials also prompted the professionals to think about different materials that could be suitable for the residential care facility. They described that they started discussing how certain materials could be used in furniture, while others could be used in cuddly toys. Moreover, they noted that some materials could help dampen sound and could be placed on walls as artwork. The professionals elaborated that all materials could be used but in different ways; for example, certain materials may not be suitable for a chair, but they could be used as an art piece. Words related to values and experiences also came up, such as “luxury”, “poor”, “minimalistic” or “personal”. Here is an example of a quote on this theme:

*Yes*,* fabrics can also be used as insulation for walls. Or*,* have a big room where you isolate a corner where you can sit*,* and it will be a different atmosphere and sound. (Nursing assistant*,* G)*

#### The uniqueness of the method in creating shared agency and development

The art and design workshops supported the construction of shared agency through the co-creation and participation of the professionals in developing concrete examples of improvements for the environment at the residential care facility. However, some participants described that they had already used similar methods to create improvements at the residential care facility. They described previous similar experiences in developmental work with the environment, aimed at better aligning the environment with the specific needs of the older persons. Crucial to this process was having management that listened when the professionals recognised the need for adaptations based on the older persons’ requirements. Sometimes, it involved creating more space to ensure that the older persons could move around safely and comfortably. An example of this is:

*We are working on it. We have tried many things for adaptation. We are still well on our way. I still think that’s positive. (Nursing assistant*,* F)*

## Discussion

Earlier research on co-creation and participation of social care professionals highlights the importance of focusing on what characterises: (a) Arenas for communication and conditions supporting increased space for different perspectives and judgements by granting and shifting real influence among all parties involved (see, for example, [34, 35]) and (b) Shared agency [32, 33] to improve the physical environment of residential care homes. This study enhances this understanding by showing how art and design workshops could:

1. serve as effective communication arenas, supporting the creation of conditions that enable diverse perspectives and shared decision-making. This aligns with value-based care principles, which emphasise the importance of integrating social values such as well-being, satisfaction, and influence into care practices [38]. By providing a platform for social care professionals to express and discuss their experiences, our findings support the argument that value-based care benefits from processes that enhance communication and stakeholder engagement.
2. facilitate shared agency and deliberative practices to further contribute to the value-based care discourse. By involving participants in the co-creation of the physical environment, these workshops align with the value-based care goal of fostering environments that meet the needs of older persons and support social care professionals [39]. This connection demonstrates how practical applications of co-creation methods can support the development of care environments that reflect value-based principles.
3. both create supportive dialogue and engage participants in the design process through the use of visual and material tools. This approach supports the value-based care with emphasis on creating care environments that are not only functional but also responsive to the needs and preferences of both older persons and staff [45].

The findings confirm, and add knowledge to, conditions highlighted by Laustsen et al. [17]. concerning the importance of professionals’ involvement and participation in integrating knowledge to develop the environment in residential care facilities. In contrast to Laustsen et al.’s [17]. findings, the professionals in our study were afforded time for participation, and the manager at the residential care facility supported the art and design workshops. Additionally, these workshops were based on concepts, materials and language that facilitated professional engagement. Therefore, the obstacles highlighted by Laustsen et al. [17]. relating to scientific concepts and language did not arise during our workshops. Accordingly, our study responds to the call for further research on the co-creation and participation of professionals in designing the physical environment of residential care facilities.

This study’s conclusions regarding the role of art and design workshops in facilitating communication, supporting professionals, and fostering shared agency can be contrasted and discussed in relation to the findings of Berge et al.’s [5] study, which explores frail older people’s experiences and reasoning about involvement in research. While our study highlights art and design workshops as communication arenas that facilitate deliberative practices among social care professionals, Berge et al. [5] delves into how frail older people perceive their involvement in research. Despite the differences in participants and contexts, both studies touch upon power dynamics and the significance of feeling heard and valued in the research process. Discussing how power structures manifest differently in each context and how involvement in research or workshops can either address or exacerbate feelings of powerlessness would enrich the discourse.

By juxtaposing and discussing the conclusions of this study with those of Berge et al. [5], we can explore the nuanced intersections between different forms of participatory practices and their implications for enhancing communication, support, and agency among diverse populations. Additionally, acknowledging potential risks, such as tokenism or power imbalances, underscores the importance of genuine engagement and collaboration in both research and creative endeavours.

This study particularly addresses issues concerning methods aimed at overcoming challenges highlighted in earlier studies on co-creation and the participation of professionals. In line with earlier research, our study shows that an aesthetic function could stimulate a meaningful context [21–23]. Furthermore, our study confirms and expands on earlier research [5, 15, 46] on the understanding of how to design a professional-based action research project, aiming at co-creating knowledge by facilitating (1) the voicing of experiences and perspectives and (2) the integration of professionals’ voices in development of the environment in residential care facilities.

*Limitations* of the study are that the research was designed to describe the co-creation process between artists and professionals from a staff perspective within the studied setting. Therefore, the data provide insight into the professionals’ experiences but do not include other groups within that setting. Another limitation is the absence of observations. Implications for future research are that more studies are needed based on a combination of perspectives, including managers, professionals and older persons participating in art and design workshops.

## Conclusion

A conclusion drawn from this study is that art and design workshops could be used to facilitate the co-creation and participation of professionals in designing environments within residential care facilities. The study has identified important mechanisms that support co-creation, focusing on both the deliberation of voicing different experiences and the development of concrete examples for improving the physical environment. An art and design workshop as a deliberative practice is characterised by communication that creates space for professionals to share experiences and voice different experiences and perspectives. Furthermore, an art and design workshop as a developing practice is characterised by construction of shared agency through dialogue focused on the design of the physical environment in residential care facilities, using photographs, materials, and fabrics. Accordingly, this knowledge answers the call for research on how to overcome practical and methodological challenges in user and professional participation, as highlighted in other earlier studies on ageing and health.

## Electronic supplementary material

Below is the link to the electronic supplementary material.

Supplementary Material 1.

## Data Availability

The data produced and analysed in this study are not accessible to the public. This is because the participants were assured of confidentiality when they gave their informed consent. However, de-identified data can be provided by the corresponding author upon reasonable request for review purposes. These data will be securely stored at the University of Gothenburg, Sweden, for a period of 10 years from the date of publication. It is important to note that all data are subject to the Swedish Public Access to Information and Secrecy Act, and each request for access will undergo a confidentiality assessment.
