# Supplementary material for: Art and design workshops at a residential care facility – social care professionals’ experiences of co-creation and participation in designing the physical environment
Source: BMC Health Serv Res. 2024 Nov 7;24:1359. doi: 10.1186/s12913-024-11851-x (PMC11542363; doi:10.1186/s12913-024-11851-x)
Supplement: Supplementary file 1 — Supplementary Material 1. [file 12913_2024_11851_MOESM1_ESM.docx]

**Supplementary file. Question areas for the group interviews**

1. Experiences of social care professionals regarding the co-creation between an artist and social care professionals. Example of question: "Can you describe your experience of working with the artist during the co-creation process?"
2. Experiences of social care professionals regarding the results of the co-creation that took place. Example of question: "What are your thoughts on the outcomes of the co-creation?"
3. Reflections and overall comments by social care professionals. Example of question: "Do you have any reflections or overall comments on the co-creation process?"
